# Sex steroid levels and stress-related markers in pregnant and non-pregnant women and the effect of periodontal therapy

**DOI:** 10.4317/medoral.26455

**Published:** 2024-02-18

**Authors:** Ozge Gokturk, Fatma Ucan Yarkac, Fatma Avcioglu

**Affiliations:** 1 Department of Periodontology, Faculty of Dentistry, Abant Izzet Baysal University, Bolu, Turkey; 2 Department of Periodontology, Faculty of Dentistry, Necmettin Erbakan University, Konya, Turkey; 3 Microbiology, Faculty of Medicine, Abant Izzet Baysal University, Bolu, Turkey

## Abstract

**Background:**

Periodontal disease during pregnancy can produce adverse events; in the current study stress was investigated as an exacerbating factors of periodontal disease. The aims of this study were to evaluate the possible associations between stress and pregnancy through scanning for gingivitis and to explore the effect of non-surgical periodontal therapy (NPT) on stress-related markers (CgA, AA, β-endorphin, DHEA, sIgA and NPY) and sex steroid levels (estrogen and progesterone) in pregnant and non-pregnant women.

**Material and Methods:**

A total of 87 subjects; 22 pregnant women with gingivitis, 25 periodontally healthy pregnant women; 22 non-pregnant women with gingivitis and 15 periodontally healthy non-pregnant women, participated in this study. Periodontal clinical measures, stress hormones and sex steroid levels were measured at baseline and following the periodontal therapy.

**Results:**

While periodontal therapy showed an improvement in salivary CgA, AA, β-endorphin, DHEA, and sIgA levels (*p*<0.05) in non-pregnant women with gingivitis; neuropeptide Y levels were found to be unaffected (*p*>0.05). There were no significant changes in salivary CgA, AA, DHEA, sIgA, and neuropeptide Y levels in pregnant women with gingivitis (*p*>0.05); however, a decrease in β-endorphin levels was observed after therapy (*p*<0.05). Pregnant women with gingivitis had higher gingival crevicular fluid (GCF) β-endorphin levels in comparison to non-pregnant women with gingivitis.

**Conclusions:**

Gingival inflammation can be a psychosocial stress inducing factor during pregnancy. Furthermore, periodontal therapy may assist in reducing stress-related hormone levels in GCF during pregnancy.

** Key words:**Gingival crevicular fluid, gingivitis, non-surgical periodontal therapy, pregnancy, saliva.

## Introduction

The increase in female sex hormone concentrations during pregnancy is assumed to have an important role in the development of gingivitis (1). Yokoyama *et al*. (2) found that both progesterone and estrogen have the potential of contributing to the progression of periodontal disease during pregnancy via increasing the production of cytokines by human gingival fibroblasts. Gürsoy *et al*. (3) reported that the estrogen levels during pregnancy are related with the degree of gingival inflammation (3). Although the influence of hormonal events on pregnancy gingivitis has been reported previously, stress may also play an important role in the progression of gingivitis in pregnant women (4). Many studies have reported an association between psychosocial stress and gingival inflammation (5,6). However, relatively few studies have focused on the role of stress in pregnancy gingivitis (7,8).

Pregnant women frequently encounter events that require adaptation throughout pregnancy and daily life. If making the required adjustments is impossible or overly difficult, psychosocial stress can occur. This stress stimulation causes induction and modulation of the hypothalamus-pituitary-adrenal (HPA) axis, ultimately resulting in the release of dehydroepiandrosterone (DHEA) from the adrenal cortex (9). Moreover, the autonomous nervous system (ANS) is induced by adrenergic receptors leading to secretion of chromogranin A (CgA) and catecholamines (adrenaline/noradrenaline) by the sensory nerve fibers and adrenal medulla when encountered with stressful events (10,11). The autonomous nervous system plays an essential role in the response of salivary glands during stress owing to adrenergic mechanisms by secreting enzymes such as salivary alpha-amylase (AA) (12,13).

Rai *et al*. (14) reported an association between periodontal disease and salivary stress markers. However, detailed information on the alterations in salivary stress markers and their relation to gingivitis during pregnancy remain unclear. The aims of the current study are to evaluate the possible associations between stress and pregnancy through scanning for gingivitis and to explore the effect of non-surgical periodontal therapy (NPT) on stress-related markers (CgA, AA, β-endorphin, DHEA, sIgA and NPY) and sex steroid levels (estrogen and progesterone) in pregnant and non-pregnant women.

## Material and Methods

- Study and Control Groups

The present study was approved by the Ethics Committee (Gaziosmapasa University Faculty of Medicine Ethics Committee) and was conducted in accordance with the Declaration of Helsinki. All subjects provided a written informed consent to participate in the study. This study was registered at Clinical Trials.gov (NCT04315532).

In total 87 patients; 22 pregnant women with gingivitis and 25 periodontally healthy pregnant women (pregnant group), 22 non-pregnant women with gingivitis and 15 periodontally healthy non-pregnant women (non-pregnant group) were recruited at the Periodontology clinic. Patients were interviewed and examined according to the eligibility criteria for inclusion in the study.

Participation criteria were being between the ages of 24-50, being pregnant for 23-30 weeks (for pregnant individuals), diagnosis of gingivitis/periodontally healthy, and not smoke. Exclusion criteria were using anti-inflammatory or antimicrobial treatment within the previous 6 months, presence of periodontitis, having systemic disease, presence of deep caries lesions, and remnant roots, and lactation.

The calibrated examiner determined the state of periodontal health and the diagnosis of gingivitis with respect to the criteria of Classification of Periodontal and Peri-Implant Diseases and Conditions of the 2017 World Workshop according to radiographic and clinical examination (15). Patients with no clinical attachment loss, no radiographic bone loss, a probing pocket depth (PPD) <3mm and bleeding on probing (BOP) at ≥10%, were diagnosed as gingivitis. Patients with no radiographic bone loss or clinical attachment loss, BOP at <10% and a PPD <3mm, were diagnosed as periodontally health.

All individuals filled out a survey form including their age, education level (primary school, secondary school, and university), oral hygiene status (frequency of tooth brushing), and income level.

- Periodontal Evaluation

Clinical periodontal examinations were performed initially (T0) and 8 weeks after the end of treatment (T8) for gingivitis patients by a calibrated examiner (FUY) using the manual periodontal probe. The examiner underwent calibration training at the onset of the study, and afterwards evaluated fifteen volunteer participants who are not related to the present study, at two separate sessions, 24 hours apart. The intra-class correlation coefficient between the PPD measurements performed at baseline and after 24 hours, was 0.94 ± 0.05. Clinical examinations of periodontally healthy pregnant individuals were performed at the first visit and 8 weeks later, while periodontally healthy non-pregnant participants were examined only at the first visit.

All teeth, excluding third molars, were examined. Plaque index (PI) and gingival index (GI) by Löe and Silness (16), PPD and BOP were measured. PPD was measured using a manual periodontal probe (O’ Probe with Williams, University of Michigan) at six sites per tooth (distobuccal, mid-buccal, mesiobuccal, distolingual, mid-lingual, and mesiolingual). The presence of BOP (occurring within 15s after periodontal probing) was assessed and scored as follows: 0 = absence of bleeding; 1 = bleeding detected.

At baseline, patients with gingivitis (N-Pr and Pr groups) received routine non-surgical periodontal therapy (including subgingival and supragingival scaling was performed via ultrasonic instruments and hand scalers) in a single session. In the control groups, when needed, initial scaling and polishing were performed to decrease pre-existing minimal gingival inflammation. Then, all participants received oral hygiene instructions.

- Collection and Analyses of Gingival Crevicular Fluid and Saliva Samples

In all groups, non-stimulated saliva was collected by expectoration for 5 minutes at T0 and T8 (7). All saliva samples were collected from all individuals between 9 am and 10 am. The participants were informed not to eat or brush their teeth in the 2 h preceding collection. After each sample was collected, it was centrifuged at 3.220 x g for 10 min. The centrifuged saliva was placed into sterile disposable tubes and stored immediately at -20°C until the day of assay. The salivary concentration of CgA, AA, β-endorphin, DHEA, sIgA, NPY, estrogen and progesterone were measured with enzyme-linked immunosorbent assay kits (ELISA) and were calculated using the standard curves constructed from each assay of salivary volume, given as pg/mL or ng/mL.

Gingival crevicular fluid (GCF) samples were collected at baseline (T0) and after therapy (T8) to assign stress marker levels in the GCF. The GCF samples were collected from randomly selected sites on non-adjacent teeth in each participant. Prior to GCF sampling, the plaque was removed from the teeth surfaces using a scaler. Afterwards, these surfaces were isolated by cotton rolls and gently dried by an air syringe. GCF was sampled with paper strips (Periopaper, ProFlow, Amityville, NY, USA) which were inserted 1 mm within the gingival crevice and left there for 30 s. Paper strips with saliva contamination or visible blood marks were discarded. The paper strips of each patient were pooled in disposable eppendorf tubes and immediately stored frozen at -20°C until laboratory analysis. On the day of analysis, the stored GCF samples were eluted using phosphate-buffered saline (PBS). The samples were left undisturbed at room temperature for 30 minutes and then tube centrifuged for 10 minutes. The eluted sample was then used in the ELISA analysis according to the manufacturer's instructions to determine the concentrations of AA, β-endorphin and DHEA.

- Statistical Analyses

The sample size was calculated based on the alteration in DHEA levels (α = 0.05, effect size d = 0.9) as a primary outcome (17). At a power of 80% and significance level of 5%, it was determined that 21 subjects per group were required. Assuming the drop-out, 24 subjects per group were enrolled. Statistical analysis was carried out using a statistical package (SPSS 21.0, SPSS Inc., Armonk, NY, USA) for clinical data. The nominal data were reported as means ± standard deviation (SD). Normality of the distribution was evaluated with the Kolmogorov-Smirnov test. In order to compare demographics of participants, *P* values were estimated by chi-square test (x2) for categorical variables. The alterations in clinical variables, the levels of GCF, salivary stress and sex steroid markers at baseline and 8 weeks after the examinations were assessed via the Kruskal-Wallis test. In addition to this, Wilcoxon rank-sums was used to investigate any significant difference in intra- group comparisons of continuous data.

## Results

- Patients’ biographical and clinical data

Demographic data of groups is included in Table 1. The majority of subjects brushed their teeth twice per day (48.8%) and had a high (university) educational level (61.9%) and high income (61.9%). There were no differences in age, educational level, income level, and frequency of tooth brushing between pregnant (Pr) and non-pregnant (N-Pr) groups (*p*>0.05).

The results of the periodontal examination of the patients are given in Table 2. Before treatment, PI and GI values were similar for N-Pr and Pr gingivitis groups (*p*> 0.05), however, the PPD value was statistically higher in Pr gingivitis group (*p*<0.05). After treatment, the periodontal parameters of Pr gingivitis group were found to be higher than N-Pr gingivitis group (*p*<0.05).

- Salivary stress-related markers’ levels

Table 3 shows the stress-related salivary markers in the Pr and N-Pr groups. While Pr gingivitis patients before treatment and the periodontally healthy group showed a significant difference in CgA, AA and β-endorphin values (*p*<0.05); there was a significant difference in CgA, AA and DHEA values between N-Pr gingivitis patients and periodontal healthy groups (*p*<0.05). Although, no differences were found between N-Pr and Pr gingivitis groups with respect to the stress markers (*p*>0.05), β-endorphin values exclusively showed a significant difference between N-Pr and Pr healthy groups (*p*<0.05). β-endorphin was found to be significantly higher in the healthy N-Pr group (*p*<0.05). After periodontal treatment, there was no statistically significant change in salivary CgA, AA, DHEA, sIgA, and NPY levels in Pr gingivitis group (*p*>0.05), with the exclusion of salivary β-endorphin levels which decreased after periodontal treatment (*p*<0.05). In the N-Pr gingivitis group, a statistically significant decrease was observed in salivary CgA, AA, β-endorphin, DHEA, and sIgA levels after periodontal therapy (*p*<0.05), with the exclusion of the NPY level which showed no alteration (*p*>0.05).

- GCF stress-related markers’ levels

The findings for the GCF stress markers of the Pr and N-Pr groups are presented in Table 4. Preceding treatment, there was a statistically significant difference in β-endorphin GCF values (*p*<0.05) between Pr gingivitis patients and the periodontally healthy groups, whereas gingivitis patients and the periodontally healthy N-Pr group showed a significant difference in AA and DHEA GCF values (*p*<0.05). Between N-Pr and Pr gingivitis groups, a statistically significant difference was observed with respect to β-endorphin GCF values (*p*<0.05). AA and DHEA GCF values showed a statistically significant difference among N-Pr and Pr healthy groups (*p*<0.05). Following periodontal treatment, β-endorphin and DHEA GCF values decreased significantly in both Pr and N-Pr gingivitis groups (*p*<0.05).

- Sex steroid markers’ levels

Table 3 demonstrates the salivary levels of sex steroids. Among the Pr groups, both estrogen and progesterone levels of patients with gingivitis were higher than the periodontally healthy group (*p*<0.05). In the N-Pr groups, it was observed that patients with gingivitis had higher estrogen levels than periodontally healthy individuals (*p*<0.05).

## Discussion

The purpose of the present study was to investigate the possible associations between stress and pregnancy through scanning for gingivitis and to explore the effect of NPT on stress-related markers and sex steroid levels in pregnant and non-pregnant women. The results of the study revealed a noTable improvement in periodontal clinical parameters after periodontal therapy in all patients. Moreover, in both Pr and N-Pr patients with gingivitis, GCF β-endorphin and DHEA levels decreased significantly following periodontal treatment. In addition to this, there was a decrease in salivary stress markers (Cg A, AA, DHEA, and β-endorphin) in N-Pr gingivitis patients after periodontal therapy, whereas the sIgA level was found to be increased.

PPD levels are prone to increase throughout pregnancy. Taani *et al*. (18) reported that pregnant women had higher PPD and GI with a similar PI when compared with non-pregnant women. Gürsoy *et al*. (1) described the progression of gingivitis in periodontally healthy pregnant women with good oral hygiene. In this study, Pr gingivitis patients were found to be similar to N-Pr gingivitis patients concerning PI and GI, while they had deeper PPD. This finding supports the prior knowledge of the presence of deeper periodontal pockets in pregnant patients. However, in the present study, GI values were found to be similar, contrary to previous studies, given that both groups of patients were diagnosed with gingivitis. In addition to this, when periodontally healthy Pr and N-Pr patients were compared, it was found that periodontally healthy Pr patients had significantly higher periodontal parameters amongst all clinical parameters. Similar results were presented by Akalın *et al*., which investigated periodontal clinical parameters in periodontally healthy pregnant women and the non-pregnant control group (19).

Pregnant women are confronted with potential stress factors, such as hormonal changes, physical alterations, and pregnancy-specific stress (e.g. fear of pain during delivery and fear of child integrity) (8). Increased stress during pregnancy may present negative influences in periodontal tissue. Furthermore, periodontal disease is another important concern causing adverse pregnancy outcomes (20). The entity of these factors renders stress and periodontal disease investigation in pregnancy to be complex, therefore this investigation requires a multidirectional notion.

Previous studies showed a close relationship between psychological stress, salivary CgA and the extent of periodontitis. Furthermore, salivary CgA and AA are noted as reliable markers pertaining to ANS activity, particularly in stressful situations (14). Sanchez *et al*. (21) noticed that periodontal inflammation provoked by stress can lead to the release of some salivary proteins such as AA. However, few studies have been published concerning the relationship between periodontal condition, pregnancy and stress. Yarkac *et al*. (7) found that the severity of gingival inflammation during pregnancy was related with stress. However, the connection between stress-related markers and the various types of periodontal diseases is yet to be confirmed. In the present study, it was observed that in both Pr and N-Pr groups, gingivitis patients had higher levels of CgA and AA in comparison to periodontally healthy patients. However, while there was a significant decrease in salivary CgA and AA levels of N-Pr gingivitis group after periodontal treatment, the Pr gingivitis group demonstrated no change. These findings support previous studies reporting that periodontal disease increases stress-related CgA and AA levels and that periodontal treatment can have a decreasing effect on the stress level (7,14).

Prior studies have revealed that salivary β-endorphin and cortisol are linked with tooth loss in the stress model (14,22). High concentrations of β-endorphin and cortisol significantly increased the expression of TIMP-1 and MMP-1, -2, -7 and -11 in gingival fibroblasts, which are the underlying actors taking part in the mechanism of enhanced periodontal destruction in correlation with psychosocial factors (23). In this study, β-endorphin levels decreased significantly after periodontal treatment in both Pr and N-Pr groups. The psychological stress cycle induces biochemical responses; β-endorphin is released first due to the initial active stress (24). At the same time, in the presence of clinical inflammation, cytokines associated with the activation of the stress system are produced. In this respect, stress and the immune system interact in the event of inflammation. Patients with gingivitis in the Pr group had significantly higher β-endorphin levels compared to the periodontally healthy, while there was no difference in the N-Pr group. In this case, the interaction between stress, periodontal inflammation and the status of pregnancy should be taken into consideration. However, as of yet, there is no published study on the relationship between β-endorphin levels, periodontal disease and pregnancy.

DHEA is affected by the dysregulation of the HPA axis and is secreted from the adrenal cortex in a similar way to cortisol (25). Though the functions of cortisol are well understood, the connection between stress-related diseases, DHEA, and the clinical outcomes of periodontal disease lack clarity. In the study where the relationship between DHEA levels and extensive periodontitis was first mentioned, Ishisaka *et al*. (9) found that the salivary levels of DHEA were associated with the severity of periodontitis. Cakmak *et al*. (17) found that salivary cortisol and DHEA levels were higher in periodontitis patients compared to healthy individuals. However, very limited information exists on the relationship between DHEA levels, pregnancy, and periodontal diseases. At present, only one study has been published having this relationship as an interest (26). Dolomatov *et al*. (26) reported that pregnant women with periodontitis were characterized by higher levels of salivary DHEA sulphate, when compared with a control group of healthy pregnant women. In the present study, although no difference was found between healthy individuals and gingivitis patients in the Pr group, salivary and GCF DHEA levels of healthy participants in N-Pr group were found to be significantly higher compared to those with gingivitis. Periodontal treatment significantly decreased GCF and salivary DHEA levels in gingivitis patients of the N-Pr group, whereas in the Pr group only GCF DHEA levels displayed a decrease. To the best of the authors’ knowledge, the present study is the first one to investigate DHEA in pregnant and non-pregnant gingivitis patients. Although, DHEA levels showed no difference between healthy individuals and gingivitis patients of the Pr group, the reason of significantly higher levels found in periodontal healthy N-Pr group may be its action of inhibiting the sulfotransferase activity of the pro-inflammatory cytokines and reducing the rates of biosynthesis.

It has been suggested that salivary IgA, which plays an important role in the local immune system of the mucosal epithelium, can be used as a possible stress marker. Additionally, it has been reported that IgA has lower secretion rates during chronic stress and negative life events in general (27). Chen *et al*. (28) found that pregnant women who did yoga to reduce stress, had lower cortisol levels and higher Ig A levels. Similar to stress, periodontal disease has a negative impact on the immune system. In the present study, while sIgA levels showed no alteration in the Pr group following periodontal treatment, they were found to be significantly increased in the N-Pr group. Periodontal therapy may reduce inflammation and improve the immune function as an anticipated response to increased sIgA levels. However, further research is required to determine the emphasis of IgA responses during periodontal treatment in pregnancy.

Neuropeptide Y is a novel neuropeptide transmitter which is co-localised with the classical neurotransmitter noradrenaline in the sympathetic nervous system. Prior studies suggest that acute stress may increase the release of NPY (29). Also, it has been found that NPY is present in GCF and displays significantly enhanced levels in healthy areas compared to those affected by periodontitis. In the study investigating NPY in human saliva in connection with periodontal disease for the first time, Haririan *et al*. (30) found a significantly higher concentration of NPY in the saliva of patients with periodontitis compared to healthy individuals. In the present research, there was no difference in salivary NPY levels between N-Pr and Pr groups. In accordance with this result, it can be inferred that periodontal treatment and/or pregnancy had no significant effect on salivary NPY levels.

Pregnancy is a period involving significant hormonal and metabolic alterations. The increased levels of sex steroids such as estrogen and progesterone can lead to adjustments in the immune response of the body, which may be in accordance with the aggravation of gingival inflammation during pregnancy (3). Gürsoy *et al*. (3) reported that the estrogen levels throughout pregnancy are related to the magnitude of gingival inflammation. However, the correlation between hormone levels during pregnancy and the severity of pregnancy gingivitis remains controversial (3). In this study, both estrogen and progesterone levels were higher in gingivitis patients compared to periodontally healthy individuals in Pr group. This result is consistent with the studies conducted by Gürsoy *et al*. (3), which suggested that high sex steroid levels increase the progression of periodontal disease. Pregnancy can be recognized as a strenuous period in a woman's life, often accompanied by anxiety and depression due to physiological, social, and emotional changes. However, the authors' inability to use a psychometric scale to evaluate stress in the participating subjects could be considered as a limitation of this study. The monitoring health or inflammation of the gingival tissues is best documented by the parameter of BOP and using this parameter as calibration training can be better for the calibration process. Therefore, calibration training was performed according to the PPD in this study, it also can be considered another limitation of the present study.

## Conclusions

We conclude that there were high stress-related hormone levels in individuals with gingival inflammation. Within the limitations of the study, it can be suggested that non-surgical periodontal therapy may assist in reducing stress-related hormone levels in saliva in non-pregnant women with gingivitis but does not improve high levels of stress-related markers in pregnancy.

## Figures and Tables

**Table 1:** Demographic data of groups.

Demographic data		Pregnant women		Non-pregnant women		p
		Gingivitis	Periodontal healthy	Gingivitis	Periodontal healthy	
Age		29.32±3.30	28.32±4.16	27.66±6.54	26.73±3.45	0.450
Education level	Primary	7 (46.7)	4 (26.7)	4 (26.7)	0 (0.0)	0.144
	Secondary	5 (29.4)	7 (41.2)	5 (29.4)	0 (0.0)	
	University	10 (19.2)	14 (26.9)	13 (25.0)	15 (28.8)	
Income	Low	3 (18.8)	3 (18.89)	5 (37.5)	4 (25.09	0.102
	Moderate	7 (43.8)	5 (31.39)	4 (25.0)	0 (0.0)	
	High	12 (23.1)	17 (32.7)	12 (23.1)	11 (21.29)	
Frequency of tooth brushing	Once a day	13 (43.3)	8 (26.7)	9 (30.0)	0 (0.0)	0.138
	Twice a day	6 (14.6)	13 (31.7)	7 (17.1)	15 (36.6)	
	Others	3 (23.1)	4 (30.8)	6 (46.2)	0 (0.0)	

**Table 2:** Median values (first and third quartiles) of and periodontal parameters of Pr and N-Pr groups, baseline (T0) and 8th week (T8).

Periodontal status		Pr group			N-Pr group			Comparisons of Pr and N-Pr groups	
		Baseline (T0)	After therapy (T8)	P^b^	Baseline (T0)	After therapy (T8)	P^b^	P^Baseline^	P^After therapy^
Plaque index	Gingivitis	1.65 (1.00-2.00)	1.00 (1.00-1.42)	0.026^*^	2.00 (1.59-3.00)	0.02 (0.00-1.00)	0.000*	0.056	0.001*
	Periodontal healthy	1.00 (0.00-2.00)	1.00 (0.00-1.00)	0.002*	0.00 (0.00-0.00)	0.00 (0.00-0.00)	1.00	0.000*	0.001*
	P^a^	0.311	0.062	-	0.000*	0.005*	-	-	-
Gingival index	Gingivitis	1.82 (1.03-2.00)	0.92 (0.34-1.02)	0.000*	2.00 (0.96-2.00)	0.01 (0.00-1.00)	0.000*	0.753	0.001*
	Periodontal healthy	1.00 (0.50-2.00)	0.40 (0.20-0.95)	0.000*	0.00 (0.00-0.00)	0.00 (0.00-0.00)	1.00	0.000*	0.000*
	P^a^	0.012*	0.054	-	0.000*	0.003*	-	-	-
Probing pocket depth	Gingivitis	2.27 (2.13-2.82)	1.74 (1.53-2.03)	0.000*	2.13 (1.88-2.27)	1.46 (1.40-1.72)	0.000*	0.046*	0.046*
	Periodontal healthy	2.14 (2.03-2.50)	1.47 (1.25-1.74)	0.000*	1.65 (1.32-2.00)	1.65 (1.32-2.00)	1.00	0.001*	0.255
	P^a^	0.172	0.039*	-	0.009*	0.227	-	-	-

p^a^= statistical difference in groups between gingivitis and periodontal healthy; p^b^= statistical difference in sub-groups (according to periodontal status) between at baseline and after therapy.

**Table 3:** Median values (first and third quartiles) of and saliva stress and sex steroid markers levels of Pr and N-Pr groups, baseline (T0) and 8th week (T8).

Periodontal status			Pr group			N-Pr group			Comparisons of Pr and N-Pr groups	
			Baseline (T0)	After therapy (T8)	P^b^	Baseline (T0)	After therapy (T8)	P^b^	P^Baseline^	P^After therapy^
Stress Markers	CgA (pg/ml)	Gingivitis	2.16 (1.98-2.42)	2.24 (1.89-2.33)	0.101	2.45 (2.08-2.58)	2.26 (2.05-2.35)	0.028*	0.071	0.685
		Periodontal healthy	2.03 (1.80-2.16)	1.75 (1.58-2.04)	0.002*	1.97 (1.71-2.34)	1.97 (1.71-2.34)	1.000	0.453	0.128
		P^a^	0.045*	0.013*	-	0.016*	0.115*	-	-	-
	sIgA (ng/ml)	Gingivitis	19.29 (16.19-21.85)	22.07 (18.54-24.84)	0.095	18.69 (15.85-22.04)	22.96 (20.21-24.17)	0.019*	0.814	0.589
		Periodontal healthy	16.53 (14.14-19.76)	16.53 (14.14-19.55)	0.068	18.49 (15.07-20.30)	18.49 (15.07-20.30)	1.000	0.426	0.426
		P^a^	0.101	0.001*	-	0.496	0.000^*^	-	-	-
	NPY (pg/ml)	Gingivitis	966.61 (677.71-1365.96)	724.68 (447.99-1167.79)	0.140	1226.56 (716.81-1710.60)	896.76 (503.75-1172.56)	0.123	0.366	0.519
		Periodontal healthy	1032.17 (790.50-1512.94)	1032.17 (790.02-1512.94)	0.109	932.33 (666.33-1196.73)	932.33 (666.33-1196.73)	1.000	0.349	0.349
		P^a^	0.631	0.042*	-	0.370	0.676	-	-	-
	AA (ng/ml)	Gingivitis	29.94 (27.02-36.77)	27.67 (23.88-39-50)	0.236	32.55 (19.44-44.49)	26.95 (18.75-30.74)	0.042*	0.907	0.241
		Periodontal healthy	18.26 (16.96-22.58)	18.26 (12.50-22.58)	0.083	17.79 (16.43-26.34)	17.79 (16.43-26.34)	1.000	0.967	0.967
		P^a^	0.000*	0.000*	-	0.028*	0.073	-	-	-
	β- endorphin (pg/ml)	Gingivitis	48.34 (43.21-64.23)	21.44 (20.20-24.83)	0.000*	50.82 (38.16-63.19)	22.96 (21.25-36.27)	0.005*	0.897	0.082
		Periodontal healthy	26.21 (24.03-35.83)	26.21 (24.03-32.61)	0.317	39.71 (29.39-51.67)	39.71 (29.39-51.67)	1.000	0.049^*^	0.023*
		P^a^	0.000*	0.001*	-	-	-	-	-	-
	DHEA (ng/ml)	Gingivitis	24.23 (19.67-29.22)	20.37 (18.24-26.69)	0.249	22.33 (20.32-26.85)	20.65 (16.94-22.96)	0.042*	0.622	0.760
		Periodontal healthy	23.99 (21.13-27.33)	24.17 (21.13-28.09)	0.317	27.14 (24.88-30.49)	27.14 (24.88-30.49)	1.000	0.204	0.067
		P^a^	0.782	0.029*	-	-	-	-	-	-
Sex Steroid Markers	Estrogen (ng/ml)	Gingivitis	701.01 (589.76-782.02)	690.86 (407.70-880.98)	0.858	259.83 (223.54-321.32)	276.95 (244.21-321.36)	0.215	0.000*	0.000*
		Periodontal healthy	480.79 (424.42-554.57)	480.79 (423.10-554.67)	0.507	229.76 (215.22-239.25)	229.76 (215.22-239.25)	1.000	0.000*	0.000*
		P^a^	0.000*	0.074	-	0.100	0.008*	-	-	-
	Progesterone (ng/ml)	Gingivitis	28.63 (24.96-32.43)	32.17 (21.44-38.77)	0.506	10.20 (6.93-18.06)	18.53 (11.10-25.91)	-	0.000*	0.001*
		Periodontal healthy	21.25 (16.98-26.52)	21.25 (18.91-28.00)	0.180	8.68 (6.93-12.80)	8.68 (6.93-12.80)	1.000	0.000*	0.000*
		P^a^	0.018*	0.024*	-	-	-	-	-	-

**Table 4:** Median values (first and third quartiles) of and GCF stress markers levels of Pr and N-Pr groups, baseline (T0) and 8th week (T8).

Periodontal status		Pr group			N-Pr group			Comparisons of Pr and N-Pr groups	
		Baseline (T0)	After therapy (T8)	P^b^	Baseline (T0)	After therapy (T8)	P^b^	P^Baseline^	P^After therapy^
AA (ng/ml)	Gingivitis	5.18 (3.36-11.68)	7.98 (3.56-10.69)	0.884	5.94 (5.16-9.13)	5.47 (4.09-7.52)	0.277	0.438	0.466
	Periodontal healthy	6.84 (5.27-9.70)	6.84 (5.27-9.70)	0.317	4.59 (3.43-5.73)	4.59 (3.43-5.73)	1.000	0.009^*^	0.006*
	P^a^	0.393	0.782	-	-	-	-	-	-
β-endorphin (pg/ml)	Gingivitis	14.58 (14.02-15.05)	13.49 (12.95-14.20)	0.019*	13.81 (13.17-14.16)	12.99 (12.65-13.21)	0.000*	0.002*	0.019*
	Periodontal healthy	13.64 (12.91-14.19)	13.64 (13.0-14.24)	0.523	13.90 (13.81-14.19)	13.90 (13.81-14.19)	1.000	0.076	0.076
	P^a^	0.000^*^	0.966	-	0.334	0.000*	-	-	-
DHEA (ng/ml)	Gingivitis	9.22 (9.08-9.55)	9.06 (8.05-9.28)	0.000*	9.14 (8.73-9.51)	8.91 (8.86-8.96)	0.000*	0.526	0.235
	Periodontal healthy	9.12 (8.67-9.42)	9.12 (8.82-9.44)	0.180	9.60 (9.25-9.86)	9.60 (9.25-9.86)	1.000	0.003*	0.013*
	P^a^	0.216	-	-	0.014*	0.000*	-	-	-

AA, α-amylase; DHEA, dehydroepiandrosterone; p^a^= statistical difference in groups between gingivitis and periodontal healthy; p^b^= statistical difference in sub-groups (according to periodontal status) between at baseline and after therapy.
